# Efficacy and Safety of Remimazolam in Short Endoscopic Procedures: A Systematic Review and Meta-Analysis

**DOI:** 10.3390/medicina61030453

**Published:** 2025-03-05

**Authors:** Yueyang Xin, Pei Lu, Shaodi Guan, Shaomeng Si, Rao Sun, Wei Xia, Hui Xu

**Affiliations:** Department of Anesthesiology and Pain Medicine, Hubei Key Laboratory of Geriatric Anesthesia and Perioperative Brain Health, and Wuhan Clinical Research Center for Geriatric Anesthesia, Tongji Hospital, Tongji Medical College, Huazhong University of Science and Technology, Wuhan 430030, China

**Keywords:** remimazolam, propofol, deep sedation, short endoscopic procedures

## Abstract

*Background and Objectives*: Appropriate sedation and anesthesia are crucial for ensuring comfortable endoscopic procedures. Propofol is one of the most often used sedatives. However, its safety and adverse effects restrict its usage. Remimazolam is a relatively new intravenous benzodiazepine that offers many benefits. Our analysis aims to evaluate the effectiveness and safety of remimazolam during short endoscopic procedures. *Materials and Methods:* We conducted a comprehensive search of the PubMed, Web of Science, ClinicalTrials.gov, and Turning Research Into Practice databases up to 31 December 2023, for randomized controlled trials published in English. Statistical analyses were performed using Cochrane Review Manager 5.4.1 and Stata Software/MP. *Results:* The success rate of sedation with remimazolam was slightly lower than that with propofol (RR: 0.99, 95% CI: 0.98~1.00; *p* = 0.004; I^2^ = 42%). As for anesthetic effect-related outcomes, remimazolam did not show advantages in onset time (MD = 12.72, 95% CI: 6.53~18.90, *p* < 0.001, I^2^ = 94%), recovery time (MD = 0.86, 95% CI: −0.55~2.27, *p* = 0.23, I^2^ = 98%), or intraoperative body movement (RR: 1.18, 95% CI: 0.60~2.32, *p* = 0.62, I^2^ = 87%). However, compared to propofol, remimazolam significantly reduced the incidence of several adverse events, including injection pain (RR: 0.07, 95% CI: 0.03~0.14, *p* < 0.001, I^2^ = 69%), intraoperative hypotension (RR: 0.38, 95% CI: 0.31~0.47, *p* < 0.001, I^2^ = 65%), bradycardia (RR: 0.25, 95% CI: 0.15~0.45, *p* < 0.001, I^2^ = 0%), and respiratory depression (RR: 0.34, 95% CI: 0.25~0.46, *p* < 0.001, I^2^ = 50%). The incidence of postoperative nausea and vomiting (PONV) was slightly higher with remimazolam (RD: 0.01, 95% CI: 0.00~0.03, *p* = 0.04, I^2^ = 33%). *Conclusions:* Remimazolam is a promising sedative for short endoscopic procedures due to its superior safety profile despite a slightly lower sedation success rate compared to propofol.

## 1. Introduction

Endoscopic procedures are becoming more and more common in clinical applications globally as a result of advancements in medicine and technology. These benefits include being less invasive, having fewer adverse responses, and having a high success rate for diagnosis and treatment [1,2,3]. In the United States alone, more than 18 million endoscopic procedures are performed annually for both diagnostic and therapeutic purposes, including bronchoscopies, hysteroscopies, and digestive endoscopies [4,5,6]. Despite their benefits, endoscopic procedures are still invasive and can cause patients to experience tension, anxiety, fear, discomfort, and even pain [7,8,9]. Effective sedation and anesthesia are, therefore, essential to alleviate patient anxiety, reduce procedural risks, and enhance patient tolerance and satisfaction [10].

Propofol is one of the most commonly used sedatives during endoscopic procedures due to its short half-life, rapid recovery, and low incidence of nausea and vomiting [11,12,13]. However, its use is associated with several safety concerns and side effects, which limit its broader application [14,15,16]. In addition to injection pain, cardiovascular depression-related hypotension, bradycardia, myocardial depression, respiratory depression-related hypoxia, aspiration pneumonia, and even the need for tracheal intubation are all possible side effects of propofol [17,18]. The American Academy of Anesthesiologists (ASA) advises against prescribing propofol to healthcare providers who are not proficient in airway management [19].

Remimazolam is a relatively novel intravenous benzodiazepine that functions as a positive allosteric modulator by interacting with the benzodiazepine binding site of the γ-aminobutyric acid subtype A (GABA_A_) receptor [20,21]. Because the structure contains a methyl propionate side chain, it has some of the pharmacological properties of remifentanil, including rapid onset, short duration of action, inactivity of metabolites, and insensitivity to infusion time [22,23,24]. In the meantime, remimazolam does not need to be cleared by the kidneys or liver but by a widely distributed enzyme called carboxylesterase, unlike midazolam, making it appropriate for individuals with renal or liver impairment [19,25,26]. Furthermore, remimazolam retains key benzodiazepine characteristics, such as water solubility, reversibility, and the absence of injection pain [22,23,24], making it especially suitable for outpatient procedures where patients can be discharged on the same day.

Given these advantages, remimazolam holds promise as an effective and safer sedative for short endoscopic procedures. To evaluate its effectiveness and safety, we conducted a meta-analysis of recent randomized controlled trials (RCTs) assessing remimazolam in the context of sedation for brief endoscopic operations.

## 2. Materials and Methods

This meta-analysis followed the PRISMA statement and registered with the International Prospective Register of Systematic Reviews (CRD42023470513). The protocol of our study is in the Supplementary Materials.

### 2.1. Data Sources and Searches

We conducted a comprehensive literature search using the PubMed, Web of Science, ClinicalTrials.gov, and Turning Research Into Practice (TRIP) databases until 31 December 2023. Only RCTs published in English would be included. We used the following search terms: ((“remimazolam” OR “CNS 7056”) AND propofol) AND (“bronchoscopy” OR “polypectomy” OR “hysteroscopy” OR “cystoscopy” OR “gastroscopy” OR “endoscopy” OR “colonoscopy”) or searched the keyword separately in ClinicalTrials.gov. Then, by rigorously and carefully screening the titles and abstracts, we eliminated literature that clearly did not meet the inclusion criteria. Finally, we reviewed the entire text and selected the literature that met the requirements based on the inclusion and exclusion criteria. This stage of work was independently completed by two authors. In cases of disagreement, the authors were advised to seek input from co-authors to reach consensus.

### 2.2. Inclusion Criteria and Exclusion Criteria

To determine study eligibility for this meta-analysis, we used the following criteria: (a) RCTs; (b) studies using remimazolam as the intervention group; (c) studies using propofol as a positive control; (d) inclusion of patients with ASA grades I, II, and III; (e) studies reporting at least the primary outcome. The exclusion criteria were as follows: (a) studies where the positive control group did not include propofol; (b) studies using only placebo as a control; (c) dose-finding studies that divided participants into different dose groups to determine the optimal dose. Two authors independently reviewed the titles and abstracts of the identified RCTs to determine their eligibility for the current investigation. The entire texts of potentially relevant studies were independently reviewed using the inclusion and exclusion criteria.

### 2.3. Outcome Measures

The primary outcome measure of this study was the success rate of sedation during brief endoscopic procedures when comparing remimazolam to propofol. The secondary outcome measures included anesthesia-related outcomes and adverse event-related outcomes. Anesthesia-related outcomes included sedation onset time, intraoperative body movement, and recovery time. Adverse events and pharmacological safety outcomes included injection pain, hypotension, bradycardia, respiratory depression, postoperative dizziness, and postoperative nausea and vomiting (PONV). The definitions of these outcomes were consistent with those used in each individual study. If multiple sedative drugs were involved, we only included data from the remimazolam and propofol groups.

### 2.4. Data Extraction and Risks of Bias Assessment

The data extracted from each included study consisted of the following information: the first author’s name, year of publication, sample size, type of procedure, patient’s age, gender, body mass index (BMI), American Society of Anesthesiologists Physical Status (ASA-PS), and dosage of remimazolam or propofol. The outcomes included those mentioned above.

Two authors independently evaluated the possibility of several biases, including allocation, performance, attrition, measurement, reporting, and overall biases, across the included RCTs using Cochrane’s technique (RoB 2) [27]. The above work was carried out by two authors and disagreements were resolved by consulting a third author.

### 2.5. Statistical Analysis

For the analysis of dichotomous outcome data, we employed the Mantel–Haenszel random-effects or fixed-effects model based on the I^2^ statistic (random-effects model: I^2^ > 50%, fixed-effects model: I^2^ ≤ 50%). Additional leave-one-out sensitivity analyses were conducted to exclude statistical outliers whenever substantial heterogeneity (I^2^ ≥ 75%) was observed [28]. The results were shown as risk difference (RD) or risk ratios (RR) with 95% confidence intervals (CIs). Continuous variable results were given as mean differences (MD) and 95% CIs. We utilized the Inverse-Variance random-effects or fixed-effects model also based on the I^2^ statistic (random-effects model: I^2^ > 50%, fixed-effects model: I^2^ ≤ 50%). If the article only provided the median and interquartile range as results, the Box–Cox transform method was employed to estimate the mean and standard deviation [29]. If multiple subgroups needed to be merged for analysis, we referred to the books of Lortie, CJ et al. [30], and Cochrane Handbook for Systematic Reviews of Interventions version 6.4 (updated August 2023), Chapter 23. For all the comparisons, two-tailed tests were performed with a significance level of *p* < 0.05. Subgroup analysis was performed based on the initial dosage of remimazolam (≤0.15 mg/kg or >0.15 mg/kg). The Cochrane Review Manager (RevMan 5.4.1; Copenhagen: The Nordic Cochrane Centre, The Cochrane Collaboration, 2014) was used for data analysis and drawing of forest plots. We assessed publication bias using a funnel plot and the Harbord test for the binary variables or the Egger test for the continuous variables [31] by the Stata Software/MP, Version 15.0 (StataCorporation, College Station, TX, USA). A *p*-value of less than 0.05 indicated the potential existence of publication bias.

## 3. Results

### 3.1. Literature Retrieval

A total of 137 articles were identified through the electronic database search based on the search criteria: 29 articles from PubMed, 46 articles from Web of Science, 21 items from ClinicalTrials.gov, and 41 articles from TRIP. After eliminating duplicates, 68 publications were reviewed.

**Figure 1:**
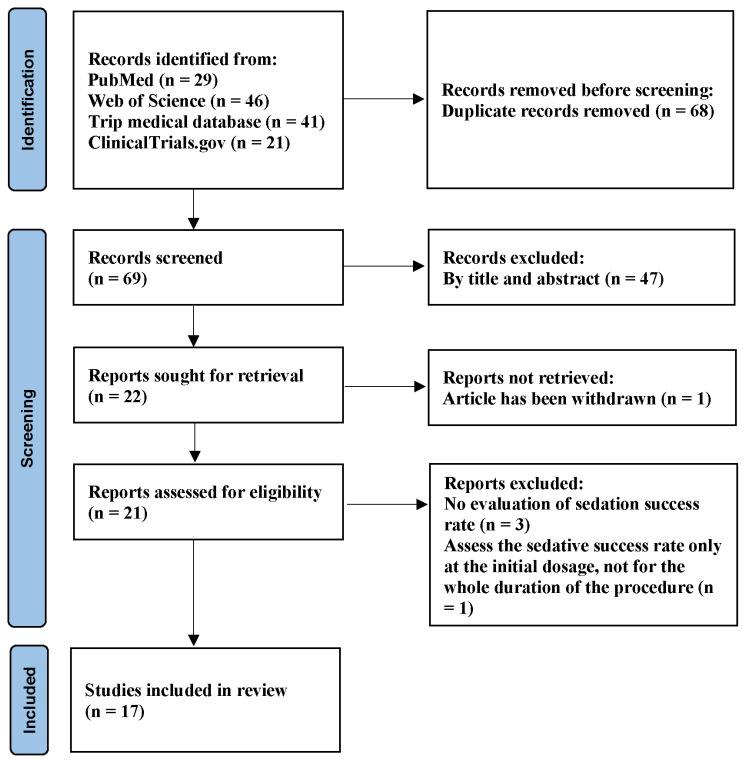
PRISMA flow plot of study selection for the current meta-analysis.

Based on the titles and abstracts, 47 papers were excluded, and 1 paper was withdrawn. Consequently, 21 potentially relevant studies were identified, and their full texts were obtained for further review. Upon full-text examination, three studies were excluded as they did not assess the success rate of sedation, and one study focused only on the success rate during the induction stage rather than the entire procedure. Ultimately, this meta-analysis included 17 articles that satisfied the predetermined inclusion criteria regarding both content and methodology. The PRISMA flowchart (Figure 1) provided a visual representation of the literature search process. The characteristics of the included studies are presented in Table 1.

### 3.2. Primary Outcome

**Figure 2:**
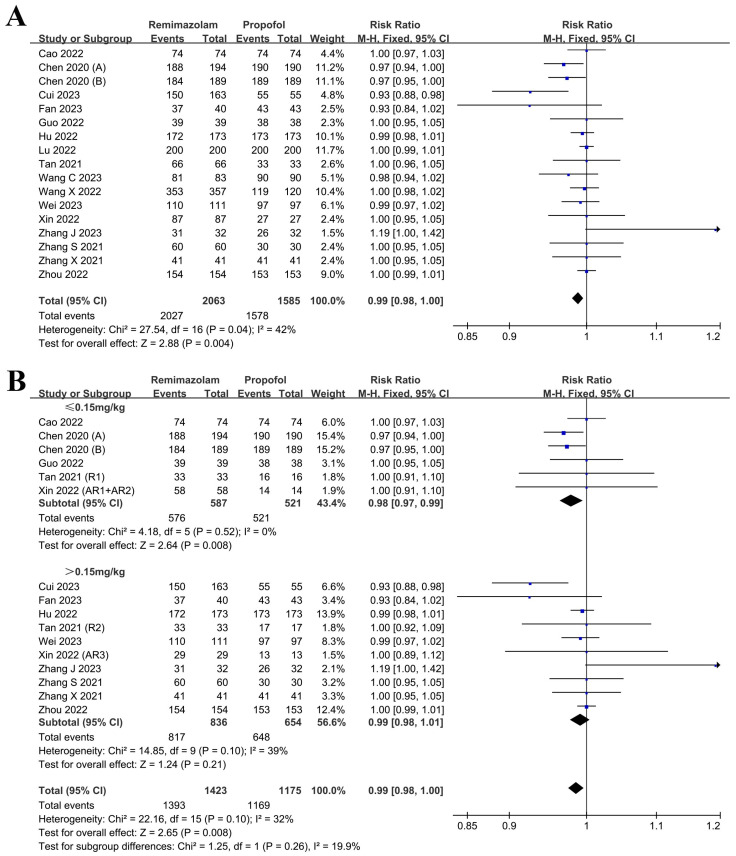
Forest plot illustrating sedation success rate and dosage subgroup analysis [1,32,33,34,35,36,37,38,39,40,41,42,43,44,45,46,47]. (**A**) sedation success rate; (**B**) dosage subgroup analysis.

The primary outcome assessed the success rate of sedation throughout the entire procedure. Although the overall efficacy of sedation with remimazolam was slightly inferior to that of propofol (RR: 0.99, 95% CI: 0.98~1.00, *p* = 0.004, I^2^: 42%, Figure 2A), both agents met clinical sedation requirements.

The results presented in Figure 2B indicate that the examination of dosage subgroups revealed a marginally reduced success rate of sedation when the initial induction dose of remimazolam was below 0.15 mg/kg compared to propofol (RR: 0.98, 95% CI: 0.97~0.99, *p* = 0.008, I^2^: 0%). However, when the initial induction dose exceeded 0.15 mg/kg, the sedation success rate of remimazolam was comparable to that of propofol (RR: 0.99, 95% CI: 0.98~1.01, *p* = 0.21, I^2^: 39%).

### 3.3. Outcomes Related to Anesthetic Effects

We next evaluated the outcomes related to anesthesia efficacy, which mainly included sedation onset time, recovery time, and intraoperative body movement.

**Figure 3:**
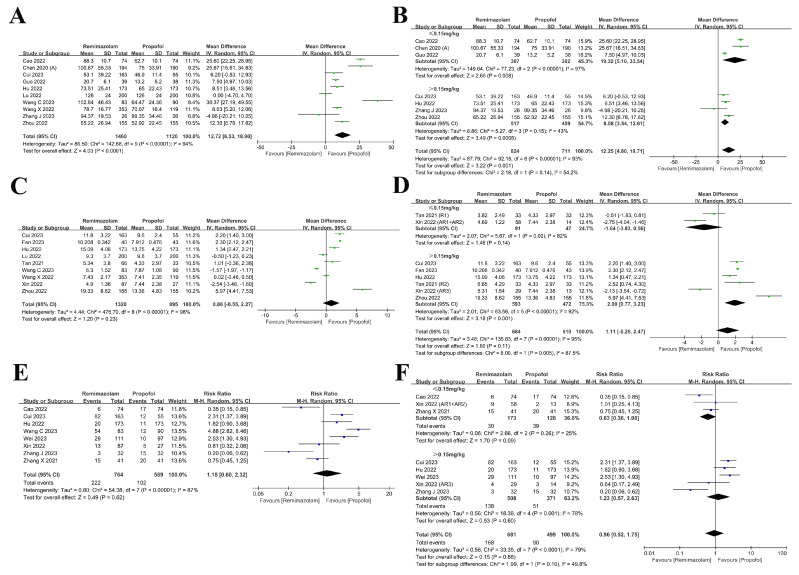
Forest plot illustrating anesthetic effect-related outcomes and dosage subgroup analysis [32,33,35,36,37,38,39,40,41,42,43,44,45,46,47]: (**A**,**B**) sedation onset time; (**C**,**D**) recovery time; (**E**,**F**) intraoperative body movement.

For ideal sedatives, rapid onset and recovery are important features [48]. A comparison was made between remimazolam and propofol regarding the sedation onset time during brief endoscopic procedures in ten studies. In contrast to propofol, remimazolam induced sedation considerably more slowly according to our analysis (MD = 12.72, 95% CI: 6.53~18.90, *p* < 0.001, I^2^: 94%, Figure 3A). The intra-dosage subgroup analysis showed that remimazolam had a longer onset time of sedation despite different initial intervention doses (≤0.15 mg/kg: MD = 19.32, 95% CI: 5.10~33.54, *p* = 0.008, I^2^: 97%; >0.15 mg/kg: MD = 8.08, 95% CI: 3.54~12.61, *p* < 0.001, Figure 3B).

As for recovery time, the results of the analysis demonstrated that the recovery time of remimazolam was not substantially shorter compared to propofol (MD = 0.86, 95% CI: −0.55~2.27, *p* = 0.23, I^2^: 98%, Figure 3C). The dosage subgroup analysis found that the recovery time with remimazolam was not significantly shorter than propofol when the starting dose of remimazolam was no more than 0.15 mg/kg (MD = −1.64, 95% CI: −3.83~0.56, *p* = 0.14, I^2^: 82%, Figure 3D). The recovery time was even longer compared with that of propofol when the initial dose of remimazolam was greater than 0.15 mg/kg (MD = 2.00, 95% CI: 0.77~3.23, *p* = 0.001, I^2^: 92%), as depicted in Figure 3D.

Intraoperative body movement as a sign of an insufficient level of sedation should be avoided as much as possible during the whole procedure [49]. Our results revealed that the incidence of body movement had no significant difference between the two groups (RR: 1.18, 95% CI: 0.60~2.32, *p* = 0.62, I^2^: 87%, Figure 3E). The analysis within the dosage subgroup showed that compared to propofol, when the induction dose of remimazolam was no more than 0.15 mg/kg, the incidence was not significantly different (RR: 0.63, 95% CI: 0.36~1.08, *p* = 0.09, I^2^: 25%, Figure 3F). When the initial dose of remimazolam exceeded 0.15 mg/kg, there was no significant difference in the occurrence of body movement compared to propofol (RR: 1.23, 95% CI: 0.57~2.63, *p* = 0.60, I^2^: 78%, Figure 3F).

The leave-one-out sensitivity analysis showed that none of the individual studies had a disproportionate impact on the pooled MD (Supplementary Figure S1A,B, Supplementary Tables S1 and S2) or RR (Supplementary Figure S1C, Supplementary Table S3) estimates. The effect size remained consistent across all the iterations, with no dependence on any single study.

### 3.4. Outcomes Pertaining to Adverse Events and Pharmacological Safety

In this section, we summarized the incidence of adverse events related to sedation, focusing on injection pain, intraoperative hypotension, bradycardia, respiratory depression, postoperative dizziness, and PONV.

**Figure 4:**
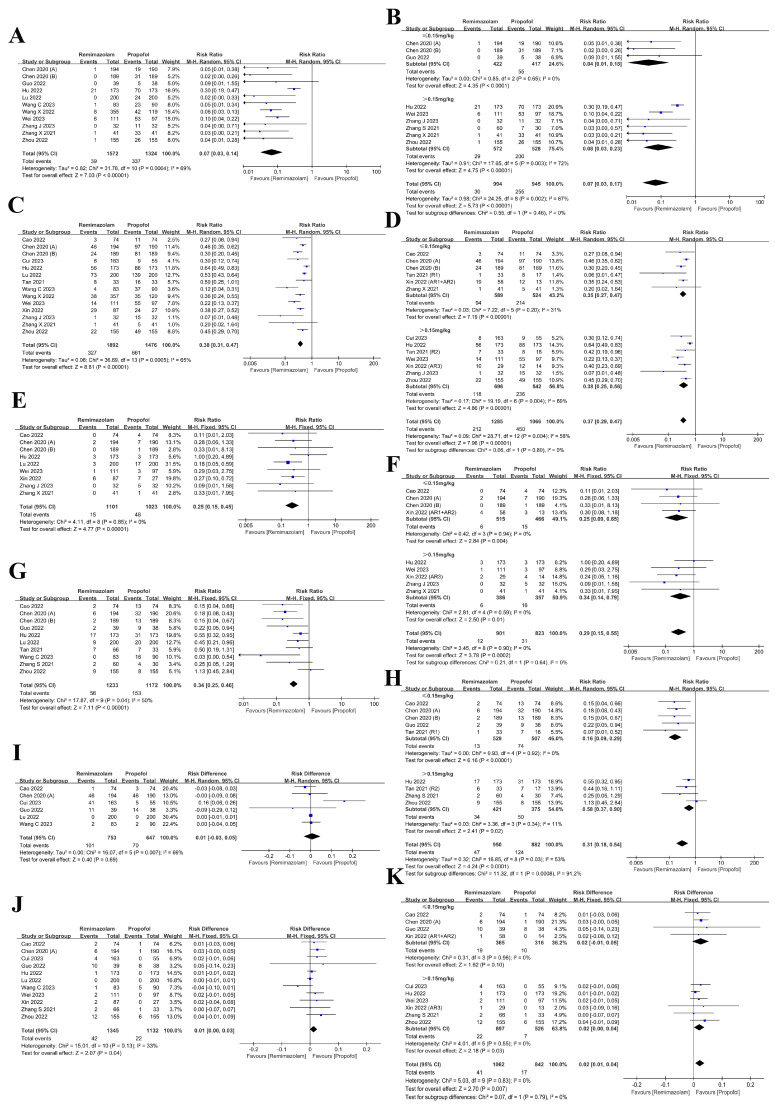
Forest plot illustrating adverse events-related outcomes and dosage subgroup analysis [1,32,33,34,35,36,37,38,39,40,41,42,43,44,46,47]: (**A**,**B**) injection pain; (**C**,**D**) hypotension; (**E**,**F**) bradycardia; (**G**,**H**) respiratory depression; (**I**) postoperative dizziness; (**J**,**K**) PONV.

We first analyzed the occurrence of injection pain in eleven trials comparing remimazolam and propofol. The results showed that remimazolam was associated with a significantly lower frequency of injection pain compared to propofol (RR: 0.07, 95% CI: 0.03~0.14, *p* < 0.001, I^2^: 69%, Figure 4A). We also assessed the incidence of injection pain in the subgroup receiving the different remimazolam induction dose. The results of the analysis revealed that remimazolam exhibited a considerable reduction in injection pain when compared to propofol irrespective of the starting dosage (≤0.15 mg/kg: RR: 0.04, 95% CI: 0.01~0.18, *p* < 0.001, I^2^: 0%; > 0.15 mg/kg: RR: 0.08, 95% CI: 0.03~0.23, *p* < 0.001, I^2^: 72%, Figure 4B).

We also examined the incidence of intraoperative hypotension, which is a known side effect of propofol due to its reduction in systemic vascular resistance [50]. Data from fourteen trials comparing remimazolam and propofol indicated that remimazolam was associated with a significantly lower incidence of hypotension (RR: 0.38, 95% CI: 0.31~0.47, *p* < 0.001, I^2^: 65%, Figure 4C). After conducting a dosage subgroup analysis, the results demonstrated that regardless of the initial dose of remimazolam, the rate of hypotension induced by remimazolam was significantly lower in comparison to propofol (≤0.15 mg/kg: RR: 0.35, 95% CI: 0.27~0.47, *p* < 0.001, I^2^: 31%; >0.15 mg/kg: RR: 0.38, 95% CI: 0.25~0.56, *p* < 0.001, I^2^: 69%, Figure 4D).

Bradycardia is also a common adverse reaction to sedatives. In our analysis of nine trials comparing the occurrence of bradycardia between remimazolam and propofol, the results showed that the incidence of bradycardia in the remimazolam group was lower compared to the propofol group throughout the sedation period (RR: 0.25, 95% CI: 0.15~0.45, *p* < 0.001, I^2^: 0%, Figure 4E). A subgroup analysis based on the induction dose of remimazolam demonstrated that a lower starting dose (≤0.15 mg/kg) was associated with a significant reduction in bradycardia compared to propofol (RR: 0.25, 95% CI: 0.09~0.65, *p* = 0.004, I^2^: 0%, Figure 4F). Similarly, a higher initial dose of remimazolam (>0.15 mg/kg) also resulted in a significant decrease in the occurrence of bradycardia compared to propofol (RR: 0.34, 95% CI: 0.14~0.79, *p* = 0.01, I^2^: 0%, Figure 4F).

Sedation results in the loss of awareness and defensive reflexes, and can lead to respiratory depression [51]. We included ten trials comparing the incidence of respiratory depression with propofol and remimazolam, which revealed that the probability of respiratory depression in the remimazolam group was substantially lower than that in the propofol group (RR: 0.34, 95% CI: 0.25~0.46, *p* < 0.001, I^2^: 50%, Figure 4G). Similarly, it was determined that the incidence of respiratory depression was considerably reduced in comparison to propofol when the initial dose of remimazolam was below 0.15 mg/kg (RR: 0.16, 95% CI: 0.09~0.29, *p* < 0.001, I^2^: 0%, Figure 4H). The occurrence of respiratory depression remained lower at doses of remimazolam exceeding 0.15 mg/kg (RR: 0.58, 95% CI: 0.37~0.90, *p* = 0.02, I^2^: 11%, Figure 4H).

Safe sedation requires postsedation care and confirmation of recovery. One of the discharge criteria is the absence of dizziness and PONV [51]. We analyzed six trials comparing the occurrence of postoperative dizziness following sedation with remimazolam and propofol. The results revealed no statistically significant difference in the occurrence of postoperative dizziness between the two groups (RD: 0.01, 95% CI: −0.03~0.05, *p* = 0.69, I^2^: 69%, Figure 4I).

In addition to hypotension, PONV is the most common adverse response to anesthesia [52]. In our analysis of eleven trials comparing the incidence of PONV between remimazolam and propofol, the results showed that the patients in the remimazolam group had a higher risk of developing PONV (RD: 0.01, 95% CI: 0.00~0.03, *p* = 0.04, I^2^: 33%, Figure 4J). The patients who used remimazolam exceeding 0.15 mg/kg for the initial dosage had a higher incidence of PONV (RD: 0.02, 95% CI: 0.00~0.04, *p* = 0.03, I^2^: 0%, Figure 4K). Nevertheless, the results of the dosage subgroup analysis displayed no statistically significant disparity in the occurrence of PONV between the remimazolam and propofol groups when the initial dosage of remimazolam was no more than 0.15 mg/kg (RD: 0.02, 95% CI: −0.01~0.05, *p* = 0.10, I^2^: 0%, Figure 4K).

### 3.5. Risk of Bias Assessment

**Figure 5:**
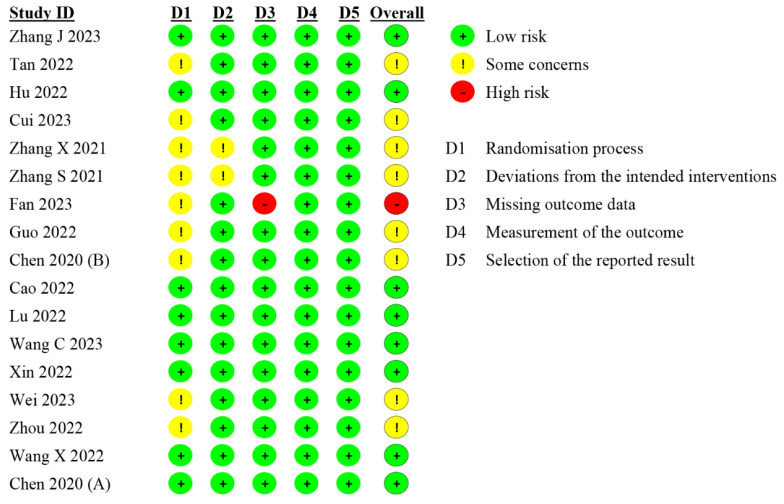
Risks of bias of the included studies [1,32,33,34,35,36,37,38,39,40,41,42,43,44,45,46,47].

During the risk of bias assessment (RoB assessment), it was found that eight out of seventeen studies were judged to have “some concerns”; moreover, one study was judged to be “high risk”, while the remaining eight trials were rated as having “low risk” (Figure 5). The reason for the “high risk” was that the author did not report the outcome completely. Nine enrollment trial’s randomization was deemed to raise “some concerns” since the assignment scheme’s description was unclear. Two studies did not indicate whether the outcome evaluator was aware of the participants’ assigned intervention.

**Figure 6:**
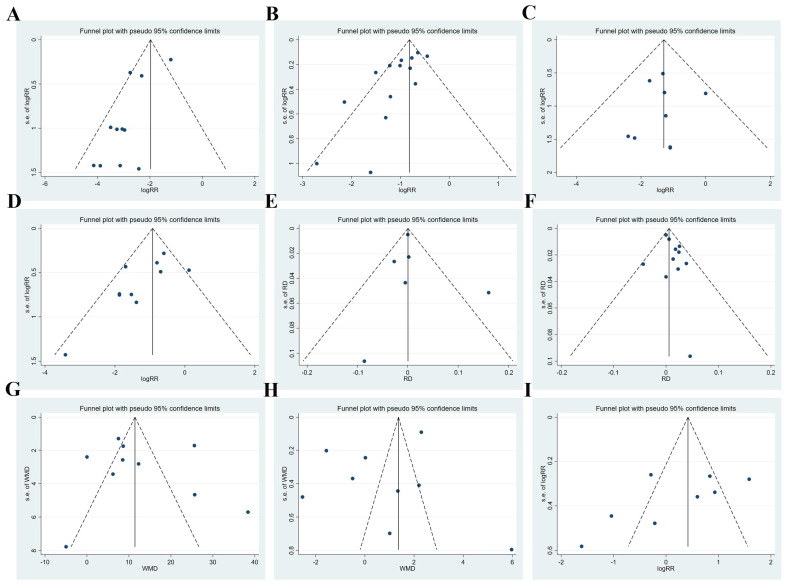
Funnel plot analyses: (**A**) injection pain; (**B**) hypotension; (**C**) bradycardia; (**D**) respiratory depression; (**E**) postoperative dizziness; (**F**) PONV; (**G**) sedation onset time; (**H**) recovery time; (**I**) intraoperative body movement. RR: risk ratio; RD: risk difference; WMD: weighted mean difference.

The publication bias revealed that, with the exception of hypotension (*p* = 0.009), all the outcomes exhibited no publication bias (*p* > 0.05). The summary of the funnel plot and tests’ details can be found in Figure 6 and Table 2.

## 4. Discussion

An ideal sedative for procedures like short endoscopies—which require light to moderate sedation—should possess characteristics such as rapid onset, precise efficacy, short duration of action, controllable recovery, and minimal impact on respiratory and circulatory systems [53]. Nowadays, propofol, α-receptor agonists like dexmedetomidine, and benzodiazepines like midazolam are commonly utilized as sedatives during short endoscopies [53,54,55]. However, each of these agents has limitations. Propofol has a narrow therapeutic index due to its potential for cardiovascular and respiratory depression, which can impede its use [19,56]. Midazolam has a lengthy half-life, roughly 1.8 to 6.4 h, and could lead to a comparatively protracted sedative effect [8]. Dexmedetomidine requires special attention because it can cause bradycardia [57].

Remimazolam, a newer benzodiazepine, has a unique molecular structure that allows it to be rapidly converted into an inactive form by carboxylesterase—an enzyme widely distributed in the body—eliminating the need for liver metabolism [26]. After administration, remimazolam plasma levels decline predictably and quickly. When administered in appropriate doses, it does not result in a prolonged sedative effect [58]. Moreover, like other benzodiazepines, its sedative effect can be reversed with flumazenil. Considering its pharmacodynamic and pharmacokinetic properties, remimazolam may be a more appropriate choice for clinical sedation in brief endoscopic procedures.

Administering anesthesia and sedation in non-operating room environments presents unique clinical challenges, requiring anesthesiologists to ensure patient safety and comfort under less controlled conditions [59]. The success rate of sedation is closely associated with procedural complications, as well as the satisfaction of both endoscopists and patients [10,60]. All the included trials met the sedation requirements for clinical endoscopic procedures and adhered to non-inferiority standards. However, our analysis revealed that the sedation success rate with remimazolam is marginally lower than that of propofol.

For safety evaluation, the occurrence and intensity of bradycardia, hypotension, and respiratory depression mostly dictate the safety of sedatives [61]. Our study indicated that overall, remimazolam was a well-tolerated sedative with fewer adverse effects than propofol no matter the different initial dosage of remimazolam. More precisely, remimazolam can lower the rate of these three abovementioned undesirable responses. Moreover, the occurrence of intraoperative hypotension and bradycardia is a predictive indicator of postoperative adverse outcomes such as acute kidney injury and postoperative cardiovascular events [62,63,64]. Therefore, reducing the occurrence of hypotension and bradycardia during short endoscopic procedures will be beneficial for the prognosis and recovery of patients. Furthermore, respiratory depression may elevate the risk of respiratory complications, particularly in elderly individuals with inadequate cardiovascular and pulmonary function reserves [39,65]. Remimazolam has a distinct advantage over propofol in reducing the occurrence of respiratory depression, making it a preferable choice for elderly patients undergoing brief endoscopic procedures.

The time required for recovery is a critical element in guaranteeing the safety and efficiency of both patients and endoscopy centers [66]. It was also reported that rapid recovery agents for colonoscopy can provide economic benefits [67]. Our findings indicated that remimazolam had a longer sedation onset time and did not offer a shorter recovery time compared to propofol. However, a recent study found that for adult patients undergoing endoscopic ultrasonography, remimazolam had a much faster induction time than propofol [68]. Additionally, for elderly patients undergoing gastrointestinal endoscopy, remimazolam significantly shortened recovery time compared to propofol [66]. These discrepancies may arise from differences in the induction dosages of remimazolam and propofol, as well as variations in patient demographics. Further clinical research is needed to better understand and clarify these findings. Our findings showed that the onset time of remimazolam was roughly 12 s longer than that of propofol. However, the existing evidence suggested that an increase in sedative onset time by approximately 10 s did not negatively impact the satisfaction of either patients or endoscopists [69,70]. Although a prolonged onset time may lengthen room turnover, it is not consistently a key determinant of endoscopic unit efficiency [71]. Future research is needed to assess how changes in sedative onset time may affect clinical satisfaction and the efficiency of endoscopic units. For intraoperative body movement, there was no significant difference between the remimazolam group and the propofol group in our study. The restricted number of included studies assessed intraoperative body movement calls for obtaining more clinical trial data to provide a further assessment of the brief endoscopies, consequently guiding our choice of the more efficient sedative.

Injection pain, postoperative dizziness, and PONV all participate in determining the sedation of patients’ experiences and feelings and, at the same time, can also be a source of stress-causing intraoperative and postoperative hypertension and tachycardia. According to our findings, the incidence of injection pain was much lower in the remimazolam group than in the propofol group, regardless of dosage. This conclusion is consistent with the findings of other studies that evaluated the safety of remimazolam [48,72]. The finding may also encourage the promotion of remimazolam in clinical settings. Additionally, our study revealed that propofol, rather than remimazolam, had more advantages in reducing the incidence of PONV, especially when the induction dosage of remimazolam is greater than 0.15 mg/kg. This finding aligns with the conclusion of a retrospective study by Suzuki et al. [73]. Postoperative outcomes, including discharge from the postanesthesia care unit (PACU), discharge home, and readmission after discharge, are all adversely affected by PONV [74]. Minimizing the occurrence of PONV in patients is also a key consideration for anesthesiologists. When the induction dose of remimazolam is higher than 0.15 mg/kg, the possible impact of PONV on patient prognosis should be taken into consideration.

From a pharmacoeconomic perspective, remimazolam was found to have a higher drug cost for intravenous anesthesia and sedation compared to propofol [75]. However, remimazolam offers two potential economic advantages that warrant further exploration. First, its safety profile allows for administration by qualified non-anesthesiologists, which could reduce personnel-related costs associated with specialized anesthesia care [75]. Second, remimazolam significantly decreased the incidence of intraoperative hypotension, a known risk factor for prolonged hospitalization and increased healthcare costs [76]. Furthermore, due to its superior airway safety, remimazolam may help mitigate the additional costs associated with airway interventions. Currently, only cost-effectiveness assessments of remimazolam in ICU patients have been conducted [77], and there is a lack of comprehensive cost-effectiveness analyses specifically focused on its perioperative application. Therefore, future health economic studies should focus on remimazolam’s cost-saving potential, particularly through reductions in cardiovascular and respiratory complications perioperatively, as well as shorter hospital stays.

There are several limitations to the current analysis. Firstly, despite conducting a dose subgroup analysis, substantial heterogeneity persisted due to variations in the administration techniques of remimazolam (weight-based versus fixed dosages) and the use of opioid medications across the included trials. Additionally, differences in gender inclusion and gender ratios are notable factors. This heterogeneity limits the robustness of our subgroup analysis and may impact the reliability of the current results. Second, given that sedation is commonly used in elderly patients undergoing diagnostic and therapeutic endoscopes, several factors contribute to the heightened sensitivity of these patients to anesthetic drugs [78,79]. These factors include reduced liver and kidney function, diminished physiological calcium storage, decreased body water content, and increased fat content. Administering the same adult dose of remimazolam may result in more deep sedation and longer recovery durations. The studies involved had notable variations in the age ranges used to define the elderly, preventing us from conducting effective age subgroup analyses (e.g., employing a consistent age range). Elderly individuals are more susceptible to various complications, necessitating additional research to figure out the efficacy and safety of remimazolam in elderly individuals. Third, Among the included studies, eight showed methodological limitations that warranted a “some concerns” rating in the risk of bias assessment. These limitations were primarily due to insufficient details on randomization procedures and the implementation of blinding protocols. Additionally, one trial did not provide comprehensive outcome reporting. While these limitations are common challenges in meta-analyses, rather than specific shortcomings of our study, they require cautious interpretation of the findings and may affect the external validity of our conclusions. This highlights the critical need for rigorously designed and well-implemented RCTs to strengthen evidence synthesis in future research. Fourth, there was publication bias for the hypotension. Similarly, enhancing the evidential quality for this outcome requires higher standards of large-sample study support.

## 5. Conclusions

In conclusion, remimazolam demonstrates potential as a sedative agent for short endoscopic procedures due to its favorable safety profile and pharmacokinetic properties. While its success rate of sedation is marginally inferior to that of propofol, remimazolam offers advantages in reducing adverse effects such as bradycardia, hypotension, and respiratory depression, expected to be more suitable for sedation in elderly patients. However, the potential for increased PONV at higher induction doses warrants caution. Further large-scale RCTs are necessary to fully establish remimazolam’s efficacy and safety across diverse patient populations and procedural contexts.

## Figures and Tables

**Table 1:** Characteristics of studies (n = 17).

	Patients (Number)	Age (Year, R vs. P)	Male/Female (R vs. P)	BMI (kg/m^2^, R vs. P)	ASA–PS	Procedures	Initial Dose/ Additional or Maintenance Dose, R	Initial Dose/ Additional or Maintenance Dose, P
Chen et al., 2020 (A) [32]	384	44.47 ± 11.67 vs. 44.43 ± 11.37	73/121 vs. 88/102	23.19 ± 2.92 vs. 23.21 ± 2.84	I–II	diagnostic or therapeutic colonoscopy	5 mg (2.5 mg)	1.5 mg/kg (0.5 mg/kg)
Chen et al., 2020 (B) [1]	378	40.78 ± 11.10 vs. 41.69 ± 10.48	78/111 vs. 69/120	23.00 ± 3.05 vs. 23.15 ± 2.88	I–II	upper gastrointestinal endoscopy	5 mg (2.5 mg)	1.5 mg/kg (0.5 mg/kg)
Zhang X et al., 2021 [33]	82	43.8 ± 8.0 vs. 45.2 ± 7.0	NA	24.7 ± 2.7 vs. 24.1 ± 2.8	I–II	hysteroscopy	0.2 mg/kg (1 mg/kg/h)	1.5–2.0 mg/kg (3.0–6.0 mg/kg/h)
Zhang S et al., 2021 [34]	90	32.60 ± 5.06, 31.13 ± 3.95 vs. 32.70 ± 5.25 *	NA	23.58 ± 3.48, 22.50 ± 2.64 vs. 23.70 ± 2.73 *	I–II	hysteroscopy	0.25 mg/kg (0.48 mg/kg/h or 0.6 mg/kg/h) *	2 mg/kg (5.0 mg/kg/h)
Tan et al., 2021 [35]	99	66.4 ± 4.8, 65.5 ± 5.2 vs. 66.2 ± 5.0 *	19/33, 22/33 vs. 21/33 *	22.7 ± 3.0, 23.4 ± 3.9 vs. 23.2 ± 3.0 *	I–II	upper gastrointestinal endoscopy	0.1 mg/kg or 0.2 mg/kg (0.05 mg/kg) *	1.0–1.5 mg/kg (0.5 mg/kg)
Cao et al., 2022 [36]	148	47.6 ± 5.8 vs. 48.5 ± 7.1	42/32 vs. 45/29	(68.5 ± 5.3 kg, 167.2 ± 5.7 cm vs. (67.7 ± 6.3 kg, 165.6 ± 5.3 cm) ^&^	II–III	gastroscopy	0.107 mg/kg ^a^	2 mg/kg ^a^
Guo et al., 2022 [37]	77	70.4 ± 3.9 vs. 69.1 ± 4.0	25/14 vs. 22/16	23.0 ± 3.0 vs. 23.0 ± 3.4	I–II	gastrointestinal endoscopy	0.15 mg/kg (0.05 mg/kg)	1.5 mg/kg (0.5 mg/kg)
Xin et al., 2022 [38]	114	54.11 ± 12.08, 49.70 ± 8.95, 52.24 ± 9.80 vs. 56.00 ± 10.13 ^b^	20/8, 23/7, 19/10 vs. 17/10 ^b^	22.20 ± 1.71, 22.43 ± 1.77, 22.40 ± 1.67 vs. 22.28 ± 1.61 ^b^	I–II	colonoscopic polypectomy	0.1 mg/kg, 0.15 mg/kg, or 0.2 mg/kg (2.5 mg) ^b^	2 mg/kg (0.66–1 mg/kg)
Hu et al., 2022 [39]	346	70.11 ± 7.37 vs. 69.92 ± 7.57	69/104 vs. 72/101	22.75 ± 3.15 vs. 22.73 ± 3.23	I–III	gastroscopy	0.2 mg/kg (0.67 mg/kg)	1.5 mg/kg (0.5 mg/kg)
Zhou et al., 2022 [40]	310	49.7 ± 13.4 vs. 51.9 ± 11.8	72/83 vs. 82/73	23.0 ± 2.62 vs. 23.5 ± 2.75	I–III	bronchoscopy	0.2 mg/kg (0.1 mg/kg)	2 mg/kg (0.75 mg/kg)
Lu et al., 2022 [41]	400	70.6 ± 4.7 vs. 70.1 ± 4.5	78/122 vs. 83/117	22.2 ± 2.5 vs. 22.2 ± 2.3	I–III	upper gastrointestinal endoscopy	300 mg/h (2.5 mg)	3.0 g/h (0.5 mg/kg)
Wang X et al., 2022 [42]	476	44.3 (33.0–54.0) vs. 46.4 (37.5–56.0) ^d^	154/203 vs. 56/63	22.82 (20.70–24.90) vs. 22.87 (21.10–24.60) ^d^	I–III	colonoscopy	7.5 mg (2.5 mg)	1.5 mg/kg (0.5 mg/kg)
Zhang J et al., 2023 [43]	66	39.06 ± 9.18 vs. 40.00 ± 10.17	32/1 vs. 31/2	27.00 ± 2.70 vs. 27.96 ± 3.22	II–III	drug-induced sleependoscopy	0.2 mg/kg (2.5 mg)	1.5 mg/kg (0.5 mg/kg)
Cui et al., 2023 [44]	218	48.9 ± 11.9, 49.6 ± 10.3, 44.6 ± 12.2 vs. 46.9 ± 11.4 ^b^	33/21, 29/25, 25/29 vs. 38/17 ^b^	24 ± 2.6, 24 ± 2.7, 23 ± 3.1 vs. 24 ± 4.6	I–II	uppergastrointestinal endoscopy	0.2 mg/kg, 0.3 mg/kg, or 0.4 mg/kg (0.05 mg/kg) ^b^	2 mg/kg (0.5 mg/kg)
Fan et al., 2023 [45]	83	43.95 ± 7.51 vs. 42.05 ± 9.071	NA	22.93 ± 3.02 vs. 23.03 ± 2.96	I–II	hysteroscopy	0.25 mg/kg (2.5 mg)	2.5 mg/kg (0.5–1 mg/kg)
Wang C et al., 2023 [46] ^c^	173	46.37 ± 12.48 vs. 49.19 ± 11.39	40/43 vs. 41/49	24.48 ± 2.36 vs. 23.72 ± 3.74	I–II	gastroscopy	7.5 mg (3.75 mg)	1.5 mg/kg (0.5 mg/kg)
Wei et al., 2023 [47] ^c^	208	44 ± 13 vs. 46 ± 13	50/61 vs. 48/49	22.8 ± 2.8 vs. 23.5 ± 2.9	I–II	uppergastrointestinal endoscopy	0.2 mg/kg (2.5 mg)	2 mg/kg (0.5 mg/kg)

*: Three patient cohorts were included, with two cohorts receiving remimazolam and one cohort receiving propofol. ^&^: Height and weight data were utilized in the study without calculating body mass index (BMI). ^a^: A 20 mg dose of propofol was employed as a rescue sedative in both the remimazolam and propofol cohorts. ^b^: Four patient cohorts were included, with three cohorts receiving remimazolam and one cohort receiving propofol. ^c^: Only data from the remimazolam and propofol groups were included in the analysis. ^d^: Data are presented as the median along with the interquartile range (IQR). ASA-PS: American Society of Anesthesiologists Physical Status; BMI: body mass index; NA: not applicable; P: propofol; R: remimazolam.

**Table 2:** Results of the Harbord test and the Egger test.

Outcomes	Methods	Coefficient	95% CI	*p*-Value
injection pain	Harbord test	−2.300	−5.601 to 1.002	0.150
hypotension	Harbord test	−2.371	−4.024 to −0.718	0.009
bradycardia	Harbord test	−0.238	−1.784 to 1.308	0.727
respiratory depression	Harbord test	−1.290	−4.651 to 2.071	0.402
postoperative dizziness	Harbord test	0.325	−2.139 to 2.789	0.733
PONV	Harbord test	0.634	−0.452 to 1.720	0.219
sedation onset time	Egger test	1.083	−5.781 to 7.948	0.725
recovery time	Egger test	−4.455	−14.074 to 5.164	0.310
intraoperative body movement	Harbord test	−6.150	−12.687 to 0.386	0.061

## Data Availability

The data supporting our study’s findings are presented in the article. The corresponding author can provide the data upon reasonable request.

## Supplementary Materials

The following supporting information can be downloaded at https://www.mdpi.com/article/10.3390/medicina61030453/s1, protocol.doc; Figure S1: Leave-one-out sensitivity analysis; Table S1: Leave-one-out sensitivity analysis detailed data for sedation onset time; Table S2: Leave-one-out sensitivity analysis detailed data for recovery time; Table S3: Leave-one-out sensitivity analysis detailed data for intraoperative body movement.

## Abbreviations

The following abbreviations are used in this manuscript:

ASA	American Academy of Anesthesiologists
GABA_A_	γ-aminobutyric acid subtype A
RCT	randomized controlled trial
BMI	body mass index
ASA-PS	American Society of Anesthesiologists Physical Status
RD	risk difference
RR	risk ratios
CI	confidence interval
MD	mean differences
PONV	postoperative nausea and vomiting
