# Supplementary material for: Efficacy and Safety of Remimazolam in Short Endoscopic Procedures: A Systematic Review and Meta-Analysis
Source: Medicina (Kaunas). 2025 Mar 5;61(3):453. doi: 10.3390/medicina61030453 (PMC11943698; doi:10.3390/medicina61030453)
Supplement: Supplementary file 1 [file medicina-61-00453-s001.zip › medicina-3478323-supplementary.pdf]

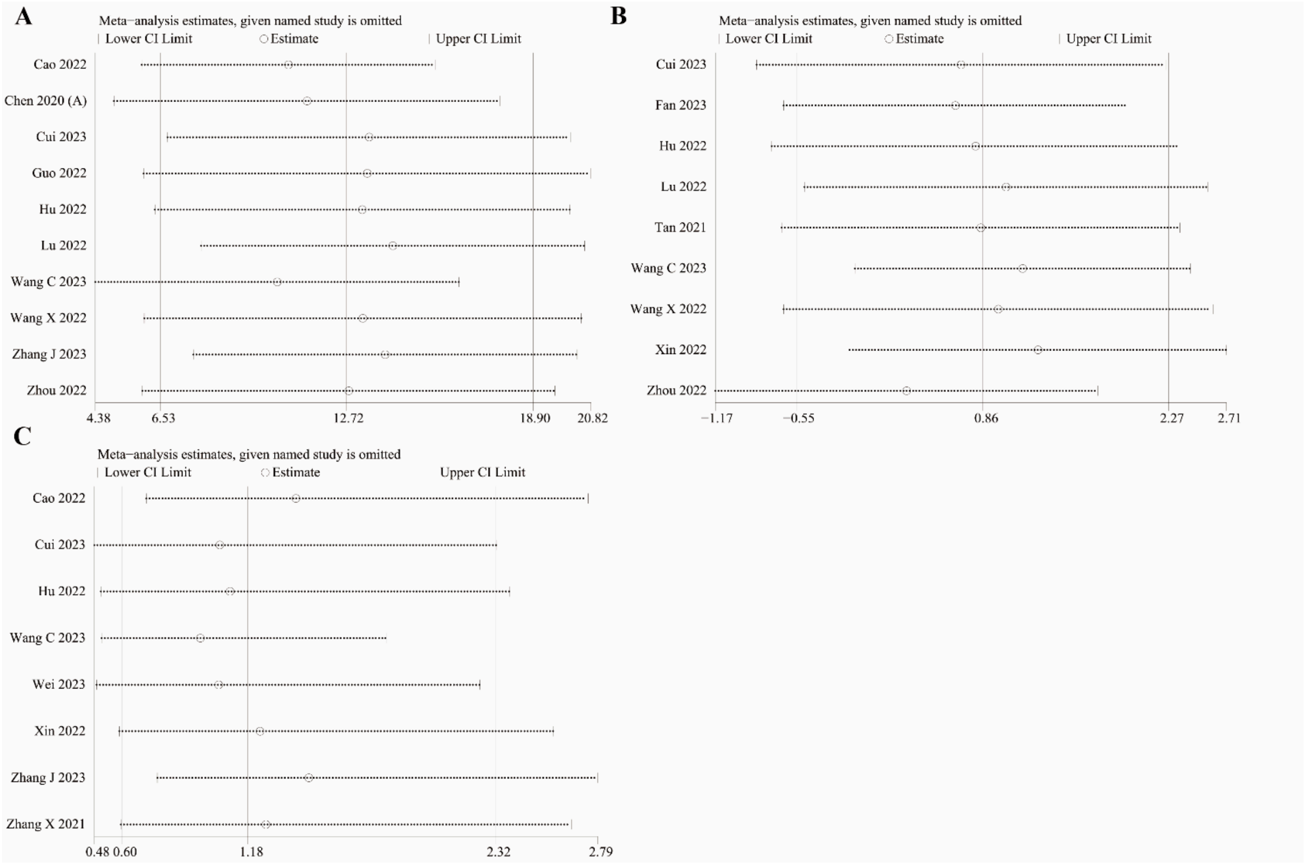

Supplementary Figure S1 Leave-one-out sensitivity analysis (A) sedation onset time; (B) recovery time; (C) intraoperative body movement.

Supplementary Table S1 Leave-one-out sensitivity analysis detailed data for sedation onset time

**Study omitted**	**Estimate (MD, sec)**	**95% CI**
Cao 2022	10.784402	5.92 to 15.65
Chen 2020 (A)	11.405547	5.00 to 17.81
Cui 2023	13.460742	6.78 to 20.14
Guo 2022	13.402665	5.98 to 20.82
Hu 2022	13.235854	6.36 to 20.11
Lu 2022	14.249451	7.89 to 20.61
Wang C 2023	10.413963	4.38 to 16.45
Wang X 2022	13.25189	6.01 to 20.50
Zhang J 2023	13.996216	7.63 to 20.36
Zhou 2022	12.782773	5.94 to 19.63
Combined	12.717377	6.53 to 18.90

CI: confidence interval, MD: mean differences

Supplementary Table S2 Leave-one-out sensitivity analysis detailed data for recovery time

Study omitted	Estimate (MD, min)	95% CI
Cui 2023	0.6956807	-0.85 to 2.25
Fan 2023	0.6512734	-0.65 to 1.95
Hu 2022	0.8044168	-0.74 to 2.35
Lu 2022	1.0362309	-0.49 to 2.57
Tan 2021	0.8447674	-0.66 to 2.36
Wang C 2023	1.1614119	-0.11 to 2.43
Wang X 2022	0.9779997	-0.65 to 2.61
Xin 2022	1.2782313	-0.15 to 2.71
Zhou 2022	0.2810020	-1.17 to 1.73
Combined	0.8607239	-0.55 to 2.27

CI: confidence interval, MD: mean differences

Supplementary Table S3 Leave-one-out sensitivity analysis detailed data for intraoperative body movement

Study omitted	Estimate (RR)	95% CI
Cao 2022	1.4040485	0.72 to 2.74
Cui 2023	1.0547579	0.48 to 2.32
Hu 2022	1.1014725	0.51 to 2.38
Wang C 2023	0.9654950	0.51 to 1.82
Wei 2023	1.0495249	0.49 to 2.25
Xin 2022	1.2394847	0.59 to 2.59
Zhang J 2023	1.4635062	0.77 to 2.79
Zhang X 2021	1.2660994	0.60 to 2.67
Combined	1.1836544	0.61 to 2.32

CI: confidence interval, RR: risk ratios
